# Haseman-Elston weighted by marker informativity

**DOI:** 10.1186/1471-2156-6-S1-S50

**Published:** 2005-12-30

**Authors:** Daniel Franke, André Kleensang, Robert C Elston, Andreas Ziegler

**Affiliations:** 1 Institut für Medizinische Biometrie und Statistik, Universität zu Lübeck, Ratzeburger Allee 160, Haus 4, 23538 Lübeck, Germany; 2 Department of Epidemiology and Biostatistics, Case Western Reserve University, Cleveland, Ohio, USA

## Abstract

In the Haseman-Elston approach the squared phenotypic difference is regressed on the proportion of alleles shared identical by descent (IBD) to map a quantitative trait to a genetic marker. In applications the IBD distribution is estimated and usually cannot be determined uniquely owing to incomplete marker information. At Genetic Analysis Workshop (GAW) 13, Jacobs et al. [*BMC Genet *2003, 4(Suppl 1):S82] proposed to improve the power of the Haseman-Elston algorithm by weighting for information available from marker genotypes. The authors did not show, however, the validity of the employed asymptotic distribution. In this paper, we use the simulated data provided for GAW 14 and show that weighting Haseman-Elston by marker information results in increased type I error rates. Specifically, we demonstrate that the number of significant findings throughout the chromosome is significantly increased with weighting schemes. Furthermore, we show that the classical Haseman-Elston method keeps its nominal significance level when applied to the same data. We therefore recommend to use Haseman-Elston with marker informativity weights only in conjunction with empirical *p*-values. Whether this approach in fact yields an increase in power needs to be investigated further.

## Background

The Haseman and Elston (HE) method [1] is the best known approach to map quantitative traits by linkage analyses. It has been extended in many different ways to increase statistical power. For example, Amos et al. [2] introduced a generalized least squares approach and weighted the squared phenotypic differences inversely proportional to their respective variances. Sham and Purcell [3] also proposed to weight phenotypes: they combined the squared trait difference and the squared mean centered sum linearly, the weights given to these two components depending on the overall trait correlation.

A different approach has been followed by Jacobs et al. [4] at the Genetic Analysis Workshop (GAW) 13. They proposed to weight families for HE linkage analyses according to marker informativity (as measured by the difference between the allele sharing at the marker and the allele sharing for a non-informative marker) and applied their method to genome scan data for blood pressure. The anticipated gain in power is substantial. For example, the *p*-value dropped from approximately 10^-2 ^to 10^-4 ^on chromosome 5 and from 10^-3 ^to 10^-5 ^on chromosome 12 for the mean systolic blood pressure. However, Jacobs et al. [4] did not show the validity of their approach, i.e., the asymptotic normality of the proposed test statistic.

Here, we pick up their work. We use the same distance metric as Jacobs et al. as well as a different simplex-based weighting scheme and show that the HE regression with weights according to this measure of marker informativity suffers from inflated type I error rates. We illustrate the effect of the weighted HE regression using data from chromosome 4 of the Aipotu population as available for GAW 14. For the analyses, we required and utilized the correct answers for the simulated data.

### Simplex weighting scheme

In the simplex weighting scheme, families are weighted according to their degree of informativity for linkage. The degree of informativity is determined by the Euclidian distance *d *between the current available identity-by-descent (IBD) marker information and the IBD information for uninformative markers.

**Figure 1:**
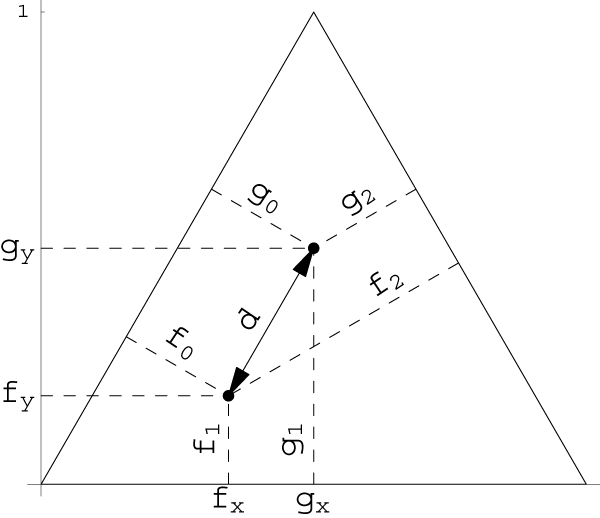
Equilateral triangle as illustration of the metric space of IBD distributions.

The calculation of the simplex weights can be illustrated by an equilateral triangle of height 1 (Fig. 1). Viviani's theorem states that, in an equilateral triangle, for any point *f *= (*f*_0_, *f*_1_, *f*_2_), the sum of the perpendiculars *f*_i _from *f *to the sides of the triangle equals the triangle's height, i.e., *f*_0 _+ *f*_1 _+ *f*_2 _= 1. Therefore, any point in Figure 1 represents exactly one possible IBD distribution. To compute the Euclidian distance  between IBD points *f *= (*f*_0_, *f*_1_, *f*_2_) and *g *= (*g*_0_, *g*_1_, *g*_2_), the IBD distributions *f *and *g *have to be mapped to the Cartesian coordinates (*f*_x_, *f*_y_) and (*g*_x_, *g*_y_), respectively. The required mapping function can easily be deduced from Figure 1 and is given by



If a genetic marker is non-informative, the IBD distribution equals (1/4,1/2,1/4). We therefore define simplex weights *w *as the Euclidian distance *d *between a marker with IBD distribution *f *= (*f*_0_, *f*_1_, *f*_2_) and a non-informative marker by



Jacobs et al. [4] proposed slightly different weights and determined marker informativity by a distance metric *D *defined as



Other measures of informativity might be preferable but are beyond the scope of this paper.

### Classical HE and weighted HE regression

For simplicity, we consider a sample of *n *independent sib pairs. Then, the classical HE regression ignores a possible dominance effect and fits the linear model

**Δ**^**2 **^= **X**_**γ **_+ *ε*, where



and the identity matrix **Ω**. The squared phenotypic difference of the *i*^th ^sib pair is represented by , and *π*_*i *_denotes the proportion of alleles IBD (*π*_*i *_= *f*_*i*2 _+ *f*_*i*1 _/ 2) of the *i*^th ^sib pair. In practice, a *t*-test statistic is employed to test the null hypothesis of no linkage. Under *H*_0 _the test statistic



is asymptotically distributed as *t*_*n*-1_, where *n *- 1 denotes the degrees of freedom. The parameter estimators for β and  are given by



As soon as weights are introduced, **Ω **no longer represents the identity matrix. Specifically, we aim to use simplex weights as described above, so that **Ω **becomes a diagonal matrix with elements *w*_*i*_(*f*). Since the IBD distribution is estimated from the available marker data,  replaces the true matrix **Ω**. Test statistics and estimates are obtained from equations (3) and (4) where **Ω **is replaced by its estimate .

## Methods

We illustrate the effect of the weighted HE regression by using data from chromosome 4 of Aipotu. The ethnic group as well as the chromosome was randomly chosen. No positive finding in the microsatellite scan can be expected for chromosome 4.

We employed a development branch of SIBPAL and GENIBD from S.A.G.E. [5] to compute three different asymptotic HE regression models for each of the 100 replicates: the classical HE method as well as HE weighted by weights according to Equations (1) and (2), respectively. GENIBD was utilized to estimate multipoint IBD distributions; estimates were obtained at the marker positions.

We wished to investigate the validity of the asymptotic distribution of the weighted HE methods. This can be achieved by testing whether the number of significant findings across a chromosome is significantly increased. For sake of simplicity, we used the 5% test level for all further investigations. For each genetic marker position, each of the 100 replicates and each weighting scheme, *p *< 0.05 was tested. Generalized estimating equations with an autoregressive (AR(1)) working correlation structure, using the replicate as class level indicator, was used for each weighting scheme to investigate whether the proportion of significant findings across the chromosome exceeded 5%. This model adequately adjusts for inter-marker correlations on a chromosome. The estimated proportion of significant findings across the whole chromosome is reported with its corresponding asymptotic 95% confidence interval (CI).

Furthermore, we want to show that the number of significant findings is greater using the weighted HE methods compared with the classical HE approach. To this end, a Wilcoxon signed rank test was employed. Specifically, we counted the number of microsatellite positions where *p <*0.05 for both weighted and the classical HE regression across the whole chromosome. The Wilcoxon signed rank tested was computed across the 100 independent chromosomes. If the weighted HE methods was too liberal, the number of significant findings would be significantly increased compared with the classical HE method.

## Results

Table 1 shows that the number of significant results at a marker position across the 100 replicates is at least as high for the weighted HE regressions as for classical HE method. This finding was substantiated by the generalized estimating equation model, which shows that the proportion of significant findings exceeds the nominal test level of 5% for both weighted HE approaches ( = 0.0685; 95% CI: 0.0546–0.0823 for the simplex weighting scheme;  = 0.0634; 95% CI: 0.0562–0.0706 for the weighting scheme proposed by Jacobs et al. [4]) but not for the classical HE method ( = 0.0552; 95% CI: 0.0431–0.0673). Furthermore, the number of markers with positive linkage is greater for both weighted HE regressions compared with the classical HE method (*p *= 3.3 × 10^-7 ^for the simplex weight (Eq. (1)), *p *= 7.1 × 10^-6 ^for the weights proposed by Jacobs et al. [4]). These findings are invariant to increased sample size as achieved by pairwise pooling of replicates (detailed results not shown).

## Conclusion

Weighting HE regression models by informativity is an appealing approach. However, some care is needed when applying this approach to real data. If phenotypes are weighted appropriately within families, this may result in substantial gain in statistical power [2,3]. Instead of weighting phenotypes, an increase in power might also be obtained by weighting according to marker informativity. This approach has been successfully utilized in the context of meta-analyses in which studies have been weighted according to their informativity [6,7]. The method of Jacobs et al. [4] combines both approaches. Jacobs et al. weighted individual sib-pair families in the HE regression according to their marker informativity as indicated above. However, the asymptotic normality of the proposed test statistic was not shown.

In this paper, using simulated data available for GAW14, we demonstrated that the HE method with family-wise weights according to marker informativity suffers from inflated type I error levels when we measure informativity as the distance between current IBD marker information and the IBD information for uninformative markers. If *p*-values are computed asymptotically from weighted regression models only, the *t*-test statistic maybe distorted.

We therefore recommend the use of weighting functions in conjunction only with empirically computed *p*-values until a theoretical solution to the detected problem is found. Furthermore, it needs to be investigated whether empirical *p *values in fact yield an increase in statistical power for weighted HE compared to the classical HE regression.

## Abbreviations

CI: Confidence interval

GAW: Genetic Analysis Workshop

HE: Haseman and Elston

IBD: Identical by descent

## Authors' contributions

AZ had the original idea for the study. DF and AK did the statistical analyses. RCE was an intellectual consultant on the study. AZ and DF wrote the report. All authors read and approved the final manuscript.

## Figures and Tables

**Table 1:** Number of one-sided *p*-values smaller than 5% in the 100 replicates of chromosome 4 from the simulated dataset of Aipotu.

	Weights		
	
Locus	HE method	Simplex weights (Eq. 1)	Jacobs et al. [4] method
D04S0128	8	9	9
D04S0129	5	6	5
D04S0130	5	6	6
D04S0131	5	5	5
D04S0132	8	9	9
D04S0133	10	12	12
D04S0134	6	7	6
D04S0135	6	9	7
D04S0136	6	6	6
D04S0137	7	7	7
D04S0138	5	6	6
D04S0139	6	7	7
D04S0140	2	3	3
D04S0141	5	6	5
D04S0142	6	8	8
D04S0143	5	5	5
D04S0144	6	9	9
D04S0145	5	7	7
D04S0146	7	9	8
D04S0147	7	8	7
D04S0148	6	8	7
D04S0149	7	9	9
D04S0150	7	10	9
D04S0151	11	12	11
D04S0152	9	11	11
D04S0153	5	7	6
D04S0154	6	8	7
D04S0155	4	4	4
D04S0156	7	8	8
D04S0157	4	5	4
D04S0158	3	3	3
D04S0159	1	1	1
D04S0160	2	2	2
D04S0161	3	4	4
D04S0162	6	6	6
D04S0163	4	5	5
D04S0164	3	5	3
D04S0165	6	10	8
D04S0166	6	10	9
D04S0167	6	6	6
D04S0168	3	4	4
D04S0169	6	6	6
D04S0170	3	4	3
D04S0171	4	7	6

## References

[B1] Haseman JK, Elston RC (1972). The investigation of linkage between a quantitative trait and a marker locus. Behav Genet.

[B2] Amos CJ, Elston RC, Wilson AF, Bailey-Wilson JE (1989). A more powerful robust sib-pair test of linkage for quantitative traits. Genetic Epidemiol.

[B3] Sham PC, Purcell S (2001). Equivalence between Haseman-Elston and variance-components linkage analyses for sib pairs. Am J Hum Genet.

[B4] Jacobs KB, Gray-McGuire C, Cartier KC, Elston RC (2003). Genome-wide linkage scan for genes affecting longitudinal trends in systolic blood pressure. BMC Genet.

[B5] (2004). S.A.G.E.: Statistical Analysis for Genetic Epidemiology. http://darwin.cwru.edu/sage/.

[B6] Loesgen S, Dempfle A, Gölla A, Bickeböller H (2001). Weighting schemes in pooled linkage analysis. Genet Epidemiol.

[B7] Dempfle A, Loesgen S (2004). Meta-analysis of linkage studies for complex diseases: an overview of methods and a simulation study. Ann Hum Genet.

